# Seroprevalence of Cytomegalovirus Infection Among HIV-Infected and HIV-Uninfected Pregnant Women Attending Antenatal Clinic in Harare, Zimbabwe

**DOI:** 10.1089/vim.2019.0024

**Published:** 2019-09-16

**Authors:** Doreen Mhandire, Kerina Duri, Mamadou Kaba, Kudakwashe Mhandire, Cuthbert Musarurwa, Emile Chimusa, Privilege Munjoma, Lovemore Mazengera, Babill Stray-Pedersen, Collet Dandara

**Affiliations:** ^1^ Division of Human Genetics, Department of Pathology, Faculty of Health Sciences, University of Cape Town, Cape Town, South Africa.; ^2^ Institute of Infectious Diseases and Molecular Medicine, University of Cape Town, Cape Town, South Africa.; ^3^ Department of Immunology, University of Zimbabwe College of Health Sciences, Harare, Zimbabwe.; ^4^ Division of Medical Microbiology, Department of Pathology, Faculty of Health Sciences, University of Cape Town, Cape Town, South Africa.; ^5^ Department of Chemical Pathology, University of Zimbabwe College of Health Sciences, Harare, Zimbabwe.; ^6^ Institute of Clinical Medicine, University of Oslo and Women's Clinic, Rikshospitalet, University Hospital, Oslo, Norway.

**Keywords:** cytomegalovirus, seroprevalence, active infection, infection reactivation, reinfection, vertical transmission

## Abstract

This study aimed to investigate the seroprevalence of cytomegalovirus (CMV) infection and risk factors associated with CMV acquisition among pregnant women in Zimbabwe. In a cross-sectional study, pregnant women were recruited in late gestation, seeking antenatal care at council clinics in three high-density suburbs in Harare, Zimbabwe. Anti-CMV IgM and IgG antibodies were quantified in serum using an enzyme-linked immunosorbent assay. Antibody avidity tests were used to distinguish active infection from viral reactivation in anti-CMV IgM-positive cases. Five hundred and twenty four women were recruited: 278 HIV infected and 246 HIV uninfected. Current or active CMV infection defined as IgM positive+low avidity was detected in 4.6% (24/524), 95% confidence interval (CI): 3–6.9 in all women, 5.8% (16/278) in the HIV infected and 3.3% (8/246), 95% CI: 1.4–6.3 in the HIV uninfected. IgG seroprevalence was 99.6% (522/524), 95% CI: 98.6–99.9 in all women. Notably, the difference in the prevalence of active CMV infection between the HIV-infected and HIV-uninfected women was not statistically significant (*p* = 0.173). The study shows a low prevalence of primary or active CMV infection among the pregnant women, but the IgG seroprevalence suggests high previous CMV exposure. Importantly, CMV seroprevalence was not associated with the HIV status of the women, perhaps due to the ubiquitous exposure of the population to CMV.

## Introduction

Cytomegalovirus (CMV) infection is endemic worldwide, with a 30–61% seroprevalence in developed countries (1,24) and 60–100% seroprevalence in developing countries (26,31,38). CMV infection is usually acquired early in life resulting in an asymptomatic, subclinical, and mostly latent infection in immune-competent persons. In the context of immune dysregulation or immune compromise such as pregnancy and HIV infection, latent CMV virus can be reactivated to cause symptomatic infection (21).

In pregnancy, reactivation of CMV predisposes to transmission of the virus from the mother to the developing fetus, leading to congenital CMV (cCMV) infection (35). Unlike other antenatal viral infections such as rubella and herpes simplex virus, prior maternal immunity to CMV fails to confer full protection from acquiring CMV infection to *in utero*, peripartum, and postpartum exposed infants (4). The consequences of cCMV can be severe and include cerebral disability, psychomotor delay, speech and language disabilities, interactive disorders, visual damage, cerebral palsy, and sensorineural hearing loss for which CMV is the leading nongenetic cause (9,25).

Previous studies have informed that the risk of vertical transmission of CMV is greater in primary CMV infection (30–50% of cases) than in latent CMV reactivation or reinfection (0.2–3% of cases) (5,17,31). However, prevalence rates of cCMV at birth are higher (∼3%) in populations with higher (nearly 100%) anti-CMV IgG seroprevalence (which indicates previous exposure to CMV) than in populations with low anti-CMV IgG seroprevalence (∼0.3%) (23,34). This discrepancy suggests that both reactivated CMV and primary CMV infection are active agents of cCMV infection. The discrepancy further elaborates on the risk of reactivation or reinfection outweighing the protective effect of maternal immunity on transplacental transmission (10).

Despite the potentially disabling consequences of CMV infection during pregnancy and the unclear role of reactivated versus primary CMV infection in cCMV, there is limited information on the prevalence of CMV infection and its associated risk factors particularly among African populations. This is despite the high burden of CMV reported in the isolated studies performed in African settings. Determining CMV infection prevalence, especially among women of childbearing age, is important in estimating the risk of cCMV infection, magnitude of burden of maternal infection, as well as identifying risk groups that could be targeted for intervention (5). The current study reports the seroprevalence of CMV in pregnant Zimbabwean women. We also investigate factors associated with CMV serostatus in HIV-infected and HIV-uninfected women recruited during late gestation from clinics in Harare, Zimbabwe.

## Methods

### Study participants

In a cross-sectional study design, pregnant women in third trimester, presenting for routine antenatal care at three council polyclinics in the high-density suburbs of Harare, were recruited from February 2016 to August 2016. Only participants who provided written informed consent for both their participation and that of their to-be-born infants were recruited. The study was granted ethical clearance by the Medical Research Council of Zimbabwe (MRCZ/A/2177) and the University of Cape Town Ethics Review Board (628/2017). All human and health research procedures were in accordance with the ethical standards of the committees responsible for human research (Institutional and National), as well as with the Helsinki Declaration of 1975, as revised in 2008. Assuming a difference of 13% in the prevalence of CMV (29) between the HIV-infected and HIV-uninfected mothers, the minimum sample size required at 5% level of significance, where *r* = 1 and 80% power is 499 mother–infant pairs.

This study was nested into the University of Zimbabwe-College of Health Sciences Birth Cohort, which aims to recruit at least 1000 well-characterized mother–infant pairs from Harare polyclinics. Participants of the birth cohort were followed up for 2 years. The Birth Cohort study aims to examine the impact on pregnancy outcomes, infant growth, immunity, and neurodevelopment of a range of factors, including coinfection with persistent viruses, maternal nutritional status, and breastfeeding, together with maternal HIV status, levels of immune suppression and immune activation, and antiretroviral therapy (ART) regimens in the HIV-infected women. This sub-study from the Birth Cohort recruited accessed a total of 527 pregnant women, 280 were HIV infected and 247 were HIV uninfected.

The current standard care for all HIV-infected pregnant women is Option B+ (ART initiation regardless of CD4^+^ T lymphocyte count or HIV viral load) to prevent mother-to-child transmission of HIV, which usually consists of TENOLAM-E (tenofovir, lamivudine, and efavirenz). The Harare polyclinics provide routine antenatal through to postnatal care for mothers and their infants residing in and around the suburbs. The setup of these polyclinics is the same as all the other public polyclinics throughout Zimbabwe.HIV-infected and HIV-uninfected pregnant women ≥28 weeks of gestational age were successfully enrolled under the University of Zimbabwe-College of Health Sciences Birth Cohort exploring HIV exposure, disease acquisition, and progression among children.

HIV-infected women were consecutively enrolled, whereas the HIV-uninfected women were systematically enrolled to include every 10th person presenting based on the approximated HIV prevalence of 10% in Zimbabwe. A pretested questionnaire was administered to collect demographic data and medical history. The HIV status of the participants was obtained from clinic records but additional confirmatory HIV testing based on the national rapid test algorithm, at the time of sample collection (39). Five milliliters of whole blood was collected in plain tubes and transported to the University of Zimbabwe, Department of Immunology laboratory where the serum was separated within 6 h of venipuncture. The serum specimens were subsequently stored at −80°C until anti-CMV analysis.

### Determination of anti-CMV serostatus

Anti-CMV IgM and IgG antibodies were detected in serum using a commercial indirect enzyme-linked immunosorbent assay (ELISA) kit (CE 0197) according to the manufacturer's instructions (EUROIMMUN, Luebeck, Germany). Participant specimens, standards, and procedural controls, provided by the manufacturer, were run simultaneously in duplicate. Extinction coefficients were determined at 450 nm. Specimens with an extinction ratio <0.8 were considered negative, extinction ratios of ≥0.8 to <1.1 were considered equivocal while extinction ratios ≥1.1 reflected positive tests for both anti-CMV IgG and IgM antibodies. Specimens with indeterminate test results were repeated and reassigned to a positive or negative group. IgG antibody titer was concurrently determined to estimate the strength or magnitude of an immune response to the CMV antigen.

Participant specimens testing positive for anti-CMV IgM were subsequently tested for IgG avidity by ELISA according to the manufacturer's instructions (EUROIMMUN). The EUROIMMUN CMV IgG avidity assay comprises two parallel assays, one with and the other without 6 M urea added to detach low-avid antibodies (37). This enables low-avid antibodies produced at the early stage of a primary infection to be distinguished from high-avid antibodies, which are characteristics of a historical infection. The avidity index generated is a ratio of the absorbance obtained from the specimen reaction to which urea buffer was added to the absorbance from the reaction without urea buffer. An avidity index ≥0.6 was considered to be a strong indicator of a primary infection dating back >3 months, whereas an index ≤0.4 indicated primary infection dating back <3 months, according to the manufacturer's protocol. An avidity index between 0.4 and 0.6 was considered equivocal (37).

### Data analysis

Study data were collected and managed using Research Electronic Data Capture (REDCap) (20). Data were analyzed using STATA version 13.1 (StataCorp, College Station, TX). Reproductive health markers as well as demographic and clinical characteristics were compared between HIV-infected and HIV-uninfected groups using the Mann–Whitney rank-sum for nonparametric variables, and chi-squared test or Fisher's exact test for categorical data. Seroprevalences were reported with their 95% confidence intervals (CIs) and compared between HIV-infected and HIV-uninfected women using chi-squared test or Fisher's exact test. A nominal *p*-value (*p* < 0.05) was considered statistically significant. Univariate logistic regression analysis was performed to investigate the association of CMV anti-IgM seropositivity with demographic and clinical factors.

## Results

### Demographic characteristics of participants

Of the 527 participants enrolled for the main birth cohort, 524 participants' (278 HIV infected and 246 HIV uninfected) samples were accessed for this study. The demographic and clinical characteristics of the participants are shown in Table 1. The median age of HIV-infected women was significantly higher (*p* < 0.001) (30 years, 25th–75th percentile: 25–34) compared with that of HIV-uninfected women (26 years, 25th–75th percentile: 21–32). The median age of gestation among the pregnant women was 33 weeks (25th–75th percentile: 29–36) and was significantly higher (*p* < 0.001) in the HIV-uninfected group (33 weeks, 25th–75th percentile: 30–36) compared with the HIV infected (32 weeks, 25th–75th percentile: 28–35). The earlier presentation of HIV-infected women to clinic (measured by lower gestation age) could be due to improved health-seeking behavior induced by HIV treatment programs.

**Table 1.** Demographic Characteristics of Study Participants

*Characteristic*	*Combined,* N* = 524*	*HIV uninfected,* n* = 246*	*HIV infected,* n* = 278*	p *(infected vs. uninfected)*
Median age in years (25th–75th percentile)	28 (23–33)	26 (21–32)	30 (25–34)	<0.001^a^
Median gestational age in weeks (25th–75th percentile)	33 (29–36)	33 (30–36)	32 (28–35)	<0.001^a^
Median partner's age in years (25th–75th percentile)	34 (29–39)	32 (27–36)	36 (31–40)	<0.001^a^
Median BMI (25th–75th percentile)	26 (24–29)	26 (23–30)	26 (24–28)	0.093^a^
Median parity (25th–75th percentile)	1 (0–2)	1 (0–2)	2 (1–3)	<0.001^a^
Median gravidity (25th–75th percentile)	2.5 (2–4)	2 (1–3)	3 (2–4)	<0.001^a^
Income (25th–75th percentile), USD/month	237.7 (150–343)	250 (150–350)	234 (150–330)	0.6329^a^
Education, *n* (%)				
Secondary	468 (89.3)	218 (88.6)	250 (89.9)	1
Primary	25 (4.8)	12 (4.88)	13 (4.7)	0.8898^b^
Tertiary	31 (5.9)	16 (6.6)	15 (5.4)	0.5867^b^
Marital status, *n* (%)				
Married	417 (79.6)	215 (87.4)	202 (72.7)	Ref.
Single	20 (3.8)	6 (2.4)	14 (5.0)	0.0596^b^
Cohabiting	83 (15.8)	25 (10.2)	58 (20.9)	0.0004^b^
Divorced	4 (0.8)	0 (0)	4 (1.4)	0.0401^b^

^a^ Mann–Whitney rank-sum test.

^b^ Chi-squared test.

BMI, body mass index.

The median parity in the study population was one child (25th–75th percentile: 0–2), but the HIV-infected women had significantly higher parity (median = 2 children, 25th–75th percentile: 1–3) compared with the HIV-uninfected women (median = 1 child, 25th–75th percentile: 0–2) (*p* < 0.001). The median gravidity in the study population was 2.5 (25th–75th percentile: 2–4). In line with the higher parity, the HIV-infected women also had significantly higher gravidity (median = 3, 25th–75th percentile: 2–4) compared with the HIV-uninfected women (median = 2, 25th–75th percentile: 1–3) (*p* < 0.001). The HIV-infected women were significantly more likely to be divorced or cohabiting than the HIV-uninfected women. Body mass index, level of education, and monthly income were comparable between the HIV-infected and HIV-uninfected women.

### Prevalence of anti-CMV antibodies

A summary of the anti-CMV antibody frequencies for this study is presented in Table 2. Of the 524 pregnant women, 522 (99.8%, 95% CI: 0.99–1) were seropositive for anti-CMV IgG. One participant from the HIV-infected group and one participant from the HIV-uninfected group tested negative for anti-CMV IgG antibodies, and both were also negative for anti-CMV IgM antibodies. In the CMV IgM test, 39 individuals (7.4%, 95% CI: 4–10) of the 524 women tested positive for CMV IgM antibodies, and there was no significant difference in the prevalence of either anti-CMV IgG or IgM seropositivity between HIV-infected and HIV-uninfected participants.

**Table 2.** Prevalence of Anti-Cytomegalovirus Antibodies in the Study Population

*Serostatus*	*All,* N* = 524*	*HIV uninfected,* n* = 246*	*HIV infected,* n* = 278*	p
IgG negative, *n* (%)	2 (0.4)	1 (0.4)	1 (0.4)	0.930^a^
IgG positive, *n* (%)	522 (99.6)	245 (99.6)	277 (99.6)	
IgM negative, *n* (%)	485 (92.6)	226 (91.9)	259 (93.2)	0.473^a^
IgM positive, *n* (%)	39 (7.4)	20 (8.1)	19 (6.8)	
IgM positive+LA, *n* (%)	24 (4.6)	8 (3.25)	16 (5.8)	0.173^a^

^a^ Chi-squared/Fisher's exact test.

LA, low avidity.

We performed CMV IgG avidity test on the 39 participants who had positive results for both anti-CMV IgG and anti-CMV IgM, and 62% (*n* = 24) showed low avidity for anti-CMV IgG antibodies while the rest showed high avidity. Interestingly, anti-CMV IgG titers were significantly higher in the HIV-infected women (median = 162.2 U/mL, interquartile range [IQR]: 120.8–200) than in the HIV-uninfected women (median = 100 U/mL, IQR: 70.4–132.1) (*p* < 0.001).

### Risk factors for CMV seropositivity

Using univariate and multivariate logistic regression analyses, none of the demographic or health characteristics (HIV status, age, parity, gestational age, level of education, and income) was significantly associated with the risk of being seropositive for either anti-CMV IgG or IgM antibodies (Table 3).

**Table 3.** Logistic Regression of Factors Associated with Anti-Cytomegalovirus Immunoglobulin M Serostatus

*Characteristic*	*Odds ratio (95% CI)*	p
HIV status	0.86 (0.42–1.75)	0.679
Age	0.97 (0.88–1.07)	0.517
Parity	0.66 (0.38–1.17)	0.154
Gravidity	1.38 (0.87–2.19)	0.172
Gestational age	1.00 (0.93–1.09)	0.906
Income	0.79 (0.47–1.33)	0.375
Education	1.23 (0.57–2.64)	0.601
Partner age	1.02 (0.94–1.09)	0.673
Marital status	0.67 (0.33–1.32)	0.252

Each of the variables was tested using univariate analysis, and no significance was observed when HIV-infected patients were compared with HIV uninfected.

CI, confidence interval.

## Discussion

We report a seroprevalence of 99.6% for anti-CMV IgG and 7.4% for IgM antibodies, in pregnant Zimbabwean women, with no significant differences in seroprevalence observed between the HIV-infected and HIV-uninfected groups. Thus, anti-CMV seropositivity was not significantly associated with HIV status in the study population. However, high anti-CMV Ig antibody titer was significantly associated with HIV positivity. There were no significant associations between anti-CMV seropositivity and demographic characteristics, such as age, parity, gravidity, level of education, and socioeconomic status. Our findings conflict with previous reports where demographic characteristics were significantly associated with either higher or lower risk of CMV infection (2,30). However, our findings are also comparable to other reports where no significant association was found between demographic characteristics and risk of CMV infection (6,18).

Understanding the epidemiology of CMV infection during pregnancy is essential for exploring control measures since cCMV infection is associated with potentially fatal and disabling effects. The high prevalence of anti-CMV IgG antibodies (99.6%) confirms reports in other studies on Egyptian, Ghanaian, Kenyan, Malawian populations and other non-African low-income countries (3,19,22,30). In contrast, lower anti-CMV IgG prevalence has been reported in the developed countries, such as the United States of America, France, and Australia (24,36). The higher prevalence of CMV infection in the developing world compared with the developed world could be explained by lower socioeconomic class characterized by overcrowded living conditions and lower income.

Markers of lower socioeconomic class have been previously reported as risk factors for CMV infection (2). Ethnicity that narrows down to genetic variation could also contribute to the differences in CMV acquisition between the developed and developing world (14,24) where the developed world mainly consists of individuals of white ethnicity while the developed world mainly consists of individuals of black ethnicity. With a 99.6% anti-CMV IgG seroprevalence, our study demonstrates an almost ubiquitous previous contact with CMV in the study population. It is possible that the participants may have acquired the infection as they were growing up since CMV acquisition usually happens during the early years of life, especially in high CMV prevalence settings (26,35).

In previous studies carried out among infants in Zimbabwe, the prevalence of CMV infection at 6 weeks of age was greater than 70%, regardless of HIV exposure, emphasizing the early acquisition of CMV infection (11,16). However, there was no report on the prevalence of maternal CMV in both studies. The high prevalence of CMV exposure in our study suggests that most of the cCMV cases in the high CMV seroprevalence populations may be due to CMV reactivation or reinfection (nonprimary CMV infection) (4). As a result, nonprimary infection would result in a higher number of cCMV cases than maternal primary infection. This also further explains the higher cases of cCMV in Africa despite the women being immune to CMV before pregnancy. The high seroprevalence of anti-CMV IgG antibodies in the study population nullified our effort to investigate the possible role of HIV in CMV acquisition.

In contrast to the nearly 100% anti-CMV IgG seroprevalence, we found a seroprevalence of 7.4% for anti-CMV IgM antibodies. Presence of anti-CMV IgM antibodies is indicative of primary infection, reactivation of previous infection, or reinfection with a different viral strain (12), but without clear demarcation. Overall, anti-CMV IgM seropositivity is confirmed with an anti-CMV IgG antibody avidity test to make a diagnosis of current CMV infection. In our case, 24/524 (4.6%) women were positive for anti-CMV IgM antibodies and had low avidity anti-CMV IgG antibodies as well. The rest of the anti-CMV IgM positives, 15/524 (2.9%) were a case of either possible reactivation of latent virus or reinfection with a different CMV viral strain.

Contrary to previous reports and the established coactivation between CMV infection and HIV infection (8,13), anti-CMV IgM seroprevalence was not significantly different between the HIV-infected and HIV-uninfected participants. The comparable seroprevalence of anti-CMV IgM antibodies between the HIV infected and HIV uninfected suggests that CMV infection is independent of HIV infection in our study population. The immune downregulation, which occurs during pregnancy, could sufficiently predispose the women to CMV infection, hence overshadow HIV infection. More importantly, the HIV-infected study participants were on ART, which in successful cases is sufficient for immune restoration, hence protection against CMV infection. However, the occurrence of CMV in some of the HIV-infected women who are also on ART may suggest differences in response to ART among the HIV-infected participants (33).

Anti-CMV IgG antibody avidity was performed on all anti-CMV IgM positive participants to confirm current infection. Antibody avidity test is the most accessible test of choice to differentiate between current and past infection especially in the resource-limited settings where polymerase chain reaction (PCR) for CMV DNA detection may not be available (28). In our study, 64% of the participants who had a positive anti-CMV IgM test had low avidity antibodies while the rest had high IgG avidity antibodies.

Our findings deviate from the study previously carried out among Egyptian pregnant women where all the participants who were positive for anti-CMV IgM antibodies had either high or intermediate avidity IgG antibodies (22). The discordance between anti-CMV IgM positivity and low avidity anti-CMV IgG antibodies results in our study could be due to prolonged circulation of anti-CMV IgM antibodies past period of active infection (7), hence an overestimation of active CMV infection cases when considering anti-CMV IgM results only. However, a study carried out among Kenyan pregnant women also reported discordance between anti-CMV IgM results and anti-CMV IgG antibodies avidity results where 80% of the individuals who were positive for anti-CMV IgM antibodies had low avidity antibodies (30).

Interestingly, anti-CMV IgG antibody titer was significantly associated with HIV status, with the HIV-infected individuals more likely to have higher anti-CMV IgG antibody titer. We suspect that since there is a massive production of IgG antibodies during a secondary immune response to a pathogen, there could be reactivation of CMV resulting in the surge of anti-CMV IgG production (27). However, due to compromised immunity especially in the HIV-infected individuals and immune senescence associated with HIV and CMV itself, there may be failure to mount an IgM immune response, sufficient to be detected by the ELISA (15). This results in underestimation of active CMV infection. In such cases, PCR to detect CMV DNA would be more useful, as it is the gold standard.

With such alarming levels of CMV exposure in pregnancy, understanding the factors associated with CMV infection will go a long way in ameliorating the burden of cCMV. However, our study did not find any significant association between the risk of being either anti-CMV IgG or anti-CMV IgM positive with either income or education. Education and income as markers of socioeconomic status have been linked to CMV infection, with lower income and lower level of education being associated with a higher risk of being anti-CMV seropositive (30,32). Zimbabwe has a literacy rate of ∼87%, ranked position 99 in the world (40), suggesting a fairly uniform and high access of written public health information. Also considering the location of the clinics where sampling was performed, the participants were most likely in the same socioeconomic group. Hence, we could not find any significant association between income and the risk of being positive for anti-CMV antibodies.

## Conclusion

In conclusion, we report a high prevalence of previous CMV exposure among women of childbearing age. Given that secondary CMV infection has been equally associated with higher cCMV to primary CMV infection in the developing countries, the finding calls for interventions to prevent vertical transmission of CMV to their offspring. CMV infection is independent of HIV status in the study population. The limitation of the study could have been the sample size where we had more HIV-infected participants than HIV-uninfected participants (278 HIV infected and 246 HIV uninfected).
